# Application of artificial intelligence in assisting treatment of gynecologic tumors: a systematic review

**DOI:** 10.1186/s42492-025-00201-1

**Published:** 2025-10-01

**Authors:** Loufei Guo, Shuaitong Zhang, Hongbo Chen, Yifu Li, Yang Liu, Wancheng Liu, Qiang Wang, Zhenchao Tang, Ping Jiang, Junjie Wang

**Affiliations:** 1 Beijing Advanced Innovation Center for Big Data-Based Precision Medicine, School of Engineering Medicine, Beihang University, Beijing, 100191 China; 2 Key Laboratory of Big Data-Based Precision Medicine (Beihang University), Ministry of Industry and Information Technology of the People’s Republic of China, Beijing, 100191 China; 3 School of Medical Technology, Beijing Institute of Technology, Beijing, 100081 China; 4 Peking University 3rd Hospital Radiation Oncology Department, Beijing, 100191 China; 5 Department of Anesthesiology, National Cancer Center/National Clinical Research Center for Cancer/Cancer Hospital, Chinese Academy of Medical Sciences and Peking Union Medical College, Beijing, 100021 China; 6 Beijing Engineering Research Center of Cardiovascular Wisdom Diagnosis and Treatment, Beijing, 100029 China

**Keywords:** Gynecologic tumors, Radiomics, Deep learning, Treatment response

## Abstract

In recent years, the application of artificial intelligence (AI) in medical image analysis has drawn increasing attention in clinical studies of gynecologic tumors. This study presents the development and prospects of AI applications to assist in the treatment of gynecological oncology. The Web of Science database was screened for articles published until August 2023. “artificial intelligence,” “deep learning,” “machine learning,” “radiomics,” “radiotherapy,” “chemoradiotherapy,” “neoadjuvant therapy,” “immunotherapy,” “gynecological malignancy,” “cervical carcinoma,” “cervical cancer,” “ovarian cancer,” “endometrial cancer,” “vulvar cancer,” “Vaginal cancer” were used as keywords. Research articles related to AI-assisted treatment of gynecological cancers were included. A total of 317 articles were retrieved based on the search strategy, and 133 were selected by applying the inclusion and exclusion criteria, including 114 on cervical cancer, 10 on endometrial cancer, and 9 on ovarian cancer. Among the included studies, 44 (33%) focused on prognosis prediction, 24 (18%) on treatment response prediction, 13 (10%) on adverse event prediction, five (4%) on dose distribution prediction, and 47 (35%) on target volume delineation. Target volume delineation and dose prediction were performed using deep Learning methods. For the prediction of treatment response, prognosis, and adverse events, 57 studies (70%) used conventional radiomics methods, 13 (16%) used deep Learning methods, 8 (10%) used spatial-related unconventional radiomics methods, and 3 (4%) used temporal-related unconventional radiomics methods. In cervical and endometrial cancers, target prediction mostly included treatment response, overall survival, recurrence, toxicity undergoing radiotherapy, lymph node metastasis, and dose distribution. For ovarian cancer, the target prediction included platinum sensitivity and postoperative complications. The majority of the studies were single-center, retrospective, and small-scale; 101 studies (76%) had single-center data, 125 studies (94%) were retrospective, and 127 studies (95%) included Less than 500 cases. The application of AI in assisting treatment in gynecological oncology remains limited. Although the results of AI in predicting the response, prognosis, adverse events, and dose distribution in gynecological oncology are superior, it is evident that there is no validation of substantial data from multiple centers for these tasks.

## Introduction

In recent years, gynecologic tumors have become a significant health concern worldwide, with a particularly high burden in many low- and middle-income cou ntries [1]. Most gynecologic tumors originate from the epithelium of the female genital tract and include cervical, ovarian, and endometrial cancers [2]. According to the World Health Organization, the global incidence and mortality rates for cervical cancers were 13.3 and 7.2 per 100,000 women-years in 2020, respectively [3]. The current standard treatment approaches for gynecologic tumors include surgery, radiation therapy, chemotherapy, and targeted therapy. Despite advancements in treatment strategies in recent decades, the prognosis of patients with gynecologic tumors remains dismal, with treatment responses varying widely among patients [4, 5]. Therefore, the identification of patients with gynecologic tumor who can benefit from these treatment strategies is essential to support clinical treatment decision-making, thereby improving the prognosis of these patients.

To address this challenge, the identification of effective and reliable prognostic indicators to identify patients likely to benefit from treatment is crucial in gynecologic oncology [6]. These indicators provide valuable insights into the treatment response and disease progression, enabling clinicians to stratify patients based on their individual risk profiles. By distinguishing between high-risk patients who may require intensified therapy and those who could benefit from treatment de-escalation, these prognostic factors play a significant role in balancing the therapeutic efficacy and minimizing treatment-related toxicity.

Currently, most prognostic indicators utilized in gynecologic tumors rely on complex multigene signatures obtained through molecular assays [7]. Common prognostic indicators include programmed cell death-1 Ligand 1, non-coding ribonucleic acids, and human epidermal growth factor receptor 2 [7]. However, this approach is costly and requires invasive procedures such as biopsy or surgical resection to obtain tumor tissue samples, limiting its clinical applicability, particularly for monitoring gynecologic tumor progression during treatment [6]. Furthermore, owing to the high heterogeneity of gynecologic tumors, the molecular characteristics derived from partial tumor tissue samples do not comprehensively represent the entire tumor [8].

In clinical practice, medical imaging examinations, such as magnetic resonance imaging (MRI), computed tomography (CT), and positron emission tomography (PET), are widely applied in the diagnosis and treatment of gynecologic tumors. These imaging examinations provide a noninvasive approach to obtain a more comprehensive characterization of gynecologic tumor [8]. Previous studies have indicated that semantic features derived from medical images, including tissue blood flow, blood volume fraction, apparent diffusion coefficient (ADC), maximum standardized uptake value, total lesion glycolysis, and metabolic tumor volume, are closely associated with the prognosis of gynecologic cancer patients [9–11]. For instance, semantic features from PET/CT have been utilized for staging cervical cancer [9]. The ADC from diffusion-weighted MRI have been used to predict disease recurrence in patients with locally advanced cervical cancer (LACC) [10]. However, the prognostic value of these imaging features remains limited.

In comparison, artificial intelligence (AI) can automatically extract high-dimensional and quantitative features from medical images to depict tumor phenotypes and correlate them with clinical outcomes [8, 12]. In contrast to molecular assays, prognostic characteristics based on AI analysis of medical images are noninvasive, compatible with the clinical workflow, and can be utilized to monitor gynecologic tumor progression during treatment [6]. In addition, AI algorithms can effectively integrate prognosis-related imaging features from various regions of interest (such as tumor areas and the surrounding tumor microenvironment) with longitudinal medical imaging data throughout the treatment process, thereby yielding more robust and powerful prognostic characteristics [13, 14].

This study provides a summary of the AI algorithms used in gynecologic tumor medical image analysis, and then presents a review of the representative applications of AI in assisting the treatment of gynecologic tumors, including predicting dose distribution, treatment response, prognosis, adverse events, and clinical target delineation of patients. Here, three prevalent gynecologic tumors are discussed: cervical, ovarian, and endometrial cancers. Finally, current challenges faced by AI in clinical translatability are summarized and the potential future directions of AI in gynecologic tumors are discussed, focusing on the future application of large models in gynecologic tumors.

## Methods

This systematic review was conducted in accordance with the Preferred Reporting Items for Systematic Reviews and Meta-Analysis (PRISMA) guidelines [15].

### Search strategy

The Literature search was conducted in the Web of Science database. The database was last screened in August 2023. The search syntax was: (TS = (artificial intelligence) OR TS = (deep learning) OR TS = (machine learning) OR TS = (radiomics)) AND (TS = (radiotherapy) OR TS = (chemoradiotherapy) OR TS = (neoadjuvant therapy) OR TS = (immunotherapy)) AND (TS = (gynecological malignancy) OR TS = (cervical carcinoma) OR TS = (cervical cancer) OR TS = (ovarian cancer) OR TS = (endometrial cancer) OR TS = (vulvar cancer) OR TS = (Vaginal cancer)).

### Study selection

**Fig. 1:**
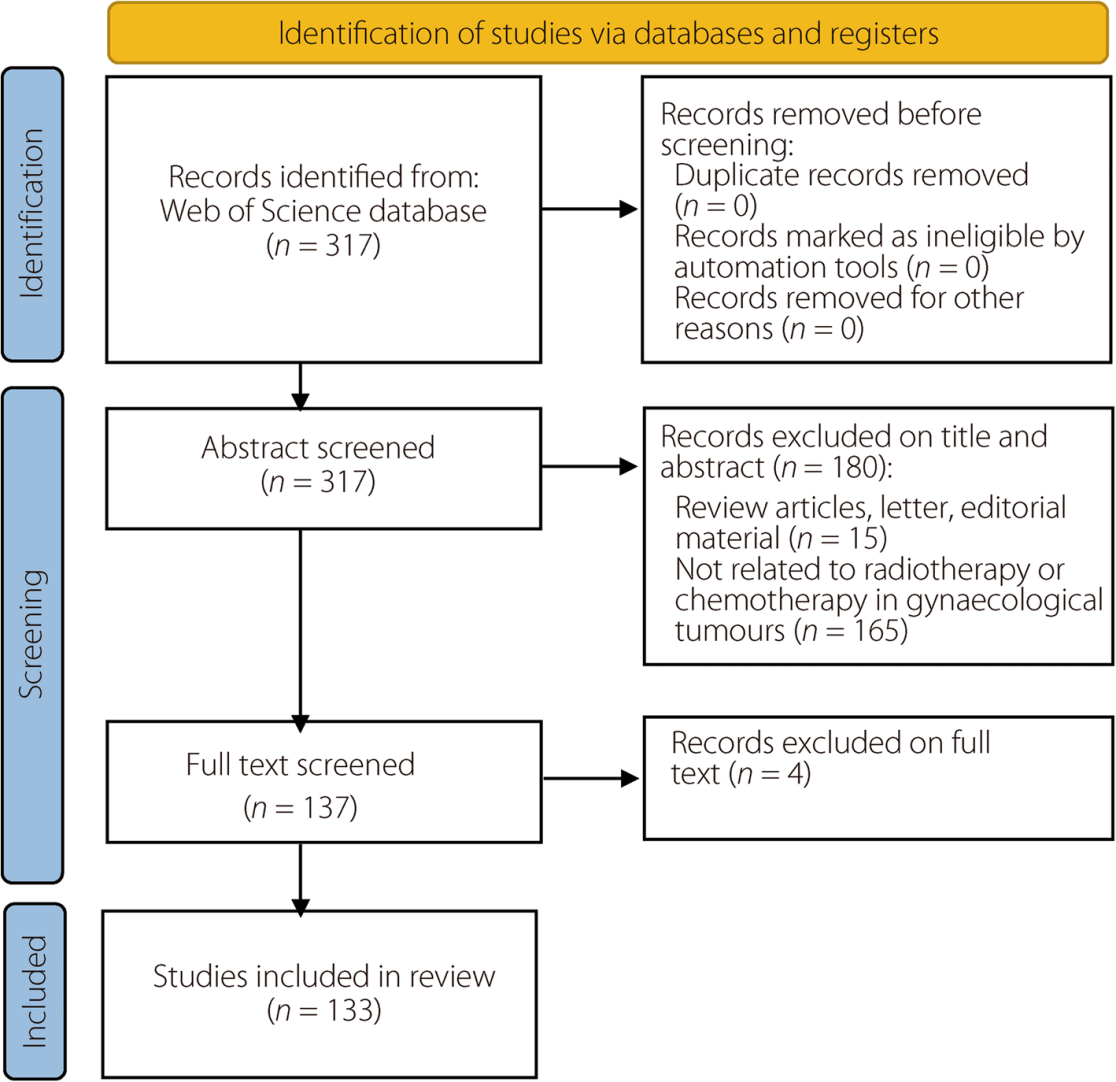
Inclusion flow diagram based on PRISMA 2020 [15]

A total of 317 articles were retrieved using the search strategy, and the titles and abstracts of these articles were independently screened by two reviewers (L.G. and Y.L.). Discrepancies were resolved by a third reviewer (S.Z.). No automated tools were used for article screening. During full-text review, some articles were found to be outside the scope of this study. Articles that used AI approaches in gynecological oncology to assist treatment were included in this systematic review. Review articles, letters, and editorials were also excluded. Articles unrelated to radiotherapy/chemotherapy, AI, and gynecological oncology were excluded. In total, 133 articles were included in the analysis. The database search and selection processes are described in the inclusion flow diagram in Fig. 1.

## AI methodology in the field of gynecologic tumors

AI refers to the use of artificial methods to achieve intelligence on computers. Over the past two decades, AI has made significant strides and is now widely used in various domains, including natural language processing (NLP) and computer vision [16, 17].

AI methods are also widely used in the field of gynecologic tumors to explore imaging-based prognostic biomarkers. Two primary AI methods are employed to predict the treatment response and prognosis of patients with gynecologic tumors: a radiomics-based approach and a deep learning-based approach. Radiomics-based approach utilizes radiomics features defined by mathematical expressions to construct prediction models using traditional machine learning classifiers. In contrast, the deep learning-based approach automatically learns the tumor phenotype from imaging data without the need to define the expression of the features beforehand. This data-driven approach simplifies the model analysis steps.

### Radiomics-based approach

**Fig. 2:**
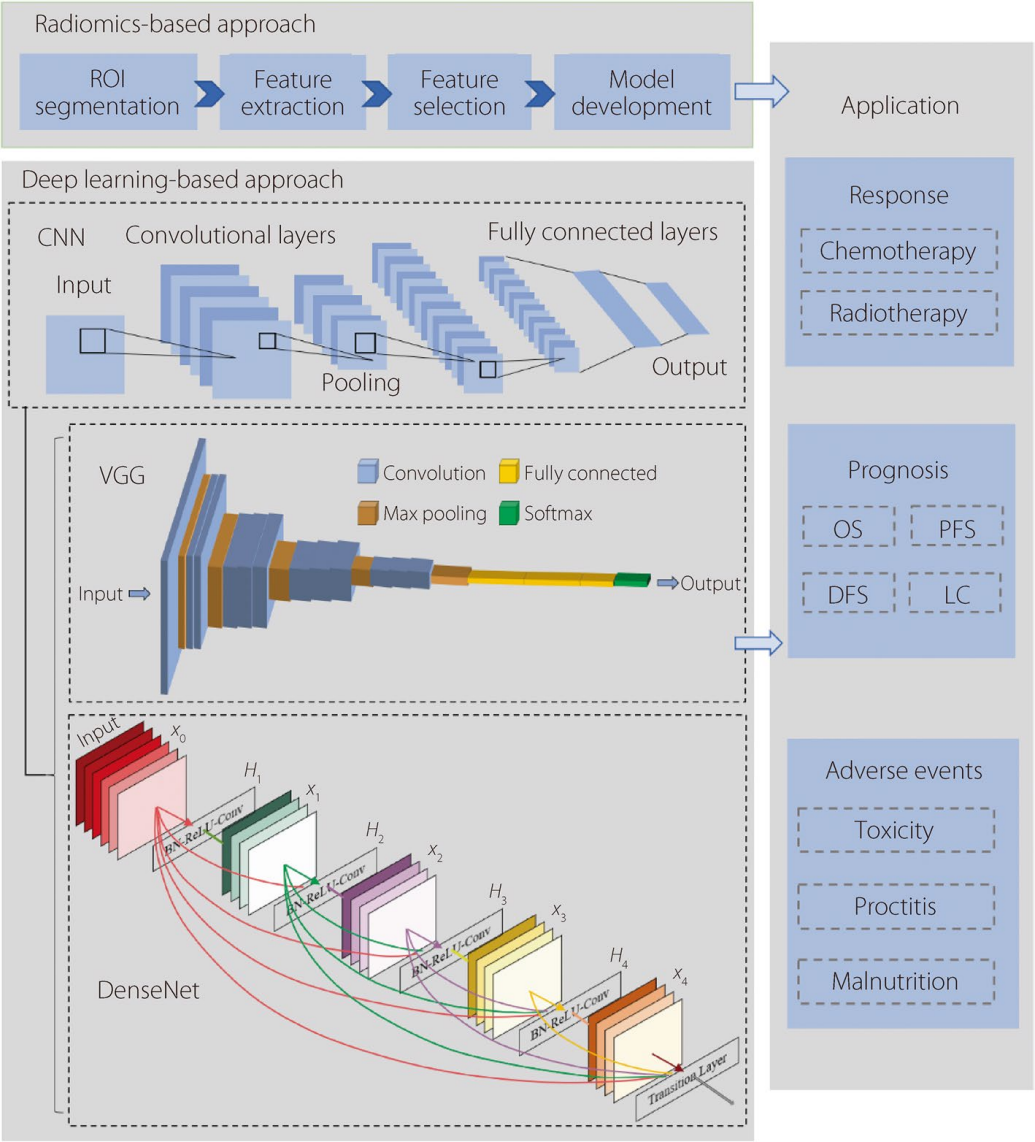
Workflow for radiomics-based approach and deep learning-based approach. Radiomics and deep learning methods can be used to predict treatment response, prognosis, and adverse events in gynecologic oncology patients. VGG and DenseNet are common CNN networks. CNN, convolutional neural network; VGG, Visual geometry group; OS, Overall survival; PFS, Progression-free survival; DFS, Disease-free survival; LC, Local control

In 2012, the concept of radiomics was initially proposed [18]. Radiomics extracts high-throughput quantitative features from various medical images using MRI, CT, PET/CT, etc. [19–21]. Radiomics has been widely used to aid in clinical diagnosis [22], predict disease prognosis [23] or adverse events [20], and define gene expression patterns [24]. Typically, a radiomics procedure (Fig. 2) includes region of interest (ROI) segmentation, feature extraction, feature selection, and model development and validation [25]. Generally, radiomics converts images into high-throughput mineable data for further analysis and utilization.

Numerous studies have focused on analyzing radiomics features extracted from the gross tumor volume (GTV) region before treatment [19]. Apart from pre-treatment medical images, images obtained during treatment also contain information associated with the treatment response and prognosis of patients. During the treatment procedure, pathophysiological changes in cancer cells, including repair, reoxygenation, redistribution, and repopulation, might occur, which makes the radiomics features from post-treatment images more likely to be associated with treatment response or prognosis. Some researchers have analyzed these unconventional radiomics features from the GTV region in medical images at different time points and found that radiomics features calculated from multiple-time medical images would improve the predictive performance of radiomics models [14, 26].

Previous studies have shown that the characteristics of the tumor-host interface can also affect treatment response and prognosis [27]. This suggests that utilizing information beyond the GTV area could potentially improve the prediction performance. Owing to tumor heterogeneity, different habitats (intratumoral and peritumoral regions) contain different features that could influence the treatment response [28]. In recent years, an increasing number of radiomics studies have focused on the prognostic value of features from multiple habitats, rather than just the features from the tumor region. Mu et al. [29] divided the tumor region on PET/CT images into four subregions according to the ^18^F-fluorodeoxyglucose (FDG) standard uptake value and analyzed radiomics features from these subregions to predict OS and PFS of LACC patients receiving radiotherapy. The radiomics model, which extracts features from multiple ROIs, exhibits excellent predictive performance.

### Deep learning-based approach

Deep learning is an important branch of machine learning that relies on interconnected neural-network layers to generate increasingly high-level representations of a given input. In the field of gynecologic tumors, deep learning methods are widely utilized in clinical applications, such as automatic target delineation [30], prediction of treatment response [31], prognosis [32], and adverse events [33].

CNNs are commonly used to analyze medical images of gynecologic tumors. CNN can automatically extract features from the medical imaging data [34]. A CNN consists of three different types of layers: convolutional, pooling, and nonlinear activation layers. In contrast to general artificial neural networks, CNNs offer three significant advantages: local connections, weight sharing, and downsampling dimensionality reduction [34]. A CNN is one of the most iconic algorithms in deep learning owing to these three advantages [34]. Commonly used CNN models include ResNet [35], VGG [36], and DenseNet [37].

### Comparison between radiomics-based approach and deep learning-based approach

Both radiomics and deep learning play important roles in analyzing the medical images of gynecologic tumors. To develop a predictive or prognostic model, radiomics usually requires well-annotated imaging data with defined clinical outcomes and a corresponding mask of the tumor as the ROI. By contrast, deep learning requires a larger amount of data, and a precise mask of the tumor is not necessary. Regarding feature extraction, radiomics directly calculates handcrafted features using explicit mathematical expressions, whereas deep learning can automatically learn task-specific features from medical imaging data. The difference in the feature extraction results is that radiomics models have better explainability [38].

## Application of AI in assisting treatment of gynecologic tumors

An increasing number of studies have reported that AI tools can extract valuable information from medical images to assist in clinical decision-making for gynecologic tumors [39–41]. This study presents a brief outline of the applications of AI approaches in several typical gynecologic tumors, including cervical, ovarian, and endometrial cancers, for predicting treatment response, prognosis, adverse events, dose distribution, and target volume delineation. Numerous papers were selected for illustration, and the characteristics of typical papers are shown in Table 1.

**Table 1:** Characteristics of radiomics studies in gynecologic cancers

Reference	Disease	Study design	No. of patients	Statistical analysis	Data	Treatment	Prediction target
Gui et al. [19]	Cervical cancer	Retrospective, multi-center study	183	Wilcoxon Mann–Whitney test, PCC, 15 different classifiers	MRI	NCRT	Response
Liu et al. [22]	Cervical cancer	Retrospective, single center study	82	RF	MRI	NCRT	Response
Tian et al. [42]	Cervical cancer	Retrospective, multi-center study	221 + 56	ICC, LASSO, SVM-RFE, ET, RF, ridge regression	CT	NACT	Response
Schernberg et al. [43]	Cervical cancer	Retrospective, single center study	69 + 39	χ^2^ and Student’s *t* tests, Pearson’s test, Cox proportional hazards model	PET	Definitive chemoradiation plus IGBAT	LC, OS
Liu et al. [44]	Cervical cancer	Retrospective, multi-center study	178 + 85	Univariate Cox analysis, PCC, LASSO, Kaplan-Meier survival analysis	MRI	CCRT	DFS
Arezzo et al. [45]	Cervical cancer	Retrospective, single center study	92	*t*-test, McNemar’s test, RFE, LR, XGBoost	MRI	NACT and radical hysterectomy	LNM
Lucia et al. [46]	Cervical cancer	Retrospective, multi-center study	52 + 50	NTCP model	Dose maps	CRT and brachytherapy	Toxicity
Wei et al. [47]	Cervical cancer	Postoperative, single center study	124 + 54	Levene’s test, *t*-test, Wilcoxon’s test, LASSO, LR	CT	Surgery and postoperative radiotherapy	Radiation proctitis
Yu et al. [48]	Cervical cancer	Retrospective, single center study	84 + 36	LASSO, ICC, LR	CT	Radiotherapy ± chemotherapy	Postoperative malnutrition
Ren et al. [49]	Cervical cancer	Retrospective, single center study	192 + 65	Wilcoxon rank-sum test, SBE-SVM	CT	IMRT	Response
Sun et al. [13]	Cervical cancer	Retrospective, multi-center study	183 + 92	χ^2^ test, RF	MRI	NACT	Response
Mu et al. [29]	Cervical cancer	Retrospective, multi-center study	78 + 76	LASSO Cox regression, Wilcoxon signed rank test, Fisher exact test, χ^2^ test, Kaplan-Meier survival analysis, log-rank test, Cox proportional hazard models	18F-FDG PET/CT	CRT	PFS, OS
Yusufaly et al. [50]	Cervical cancer	Retrospective, single center study	95 + 32	Cox regression	PET/CT	CRT and brachytherapy	Response
Wu et al. [51]	Cervical cancer	Retrospective, multi-center study	126 + 63	LASSO, LR, SVM	MRI	Radical hysterectomy and pelvic lymphadenectomy	LNM
Park et al. [52]	Cervical cancer	Retrospective, single center study	62 + 31	Random survival forest	MRI	Definitive CRT	LC, RC, DMFS, OS
Zhang et al. [14]	Cervical cancer	Retrospective, single center study	155	LASSO, LR, Cox regression	MRI	CCRT	Recurrence and DFS
Cusumano et al. [26]	Cervical cancer	Retrospective, single center study	88	Wilcoxon-Mann Whitney test, *t*-test, Shapiro-Wilk test, Benjamin-Hochberg method	MRI	CRT	Response
Bowen et al. [53]	Cervical cancer	Postoperative, single center study	21	Skillings-Mack test	MRI, PET/CT	Definitive external beam radiotherapy and brachytherapy	Response
Zhang et al. [31]	Cervical cancer	Retrospective, multi-center study	148 + 72	DenseNet-3, SVM	MRI	NACT	Response
Kawahara et al. [32]	Cervical cancer	Retrospective, single center study	63 + 26	LASSO, neural network classifiers	MRI	EBRT followed by intracavitary brachytherapy	Recurrence
Shen et al. [54]	Cervical cancer	Retrospective, single center study	142	Deep learning model	18F-FDG PET/CT	Definitive CRT	Local relapse and distant metastasis
Zhen et al. [33]	Cervical cancer	Retrospective, single center study	42	VGG-16 CNN	Rectum surface dose maps	EBRT, brachytherapy	Rectum toxicity
Cheon et al. [55]	Cervical cancer	Retrospective, single center study	199 + 82	LR, MLP, Lightweight deep learning model	Dose parameters	Definitive radiotherapy	Late bladder toxicity
Xiao et al. [56]	Cervical cancer	Retrospective, multi-center study	650	Resnet18, *t*-test, Mann-Whitney U test, χ^2^ test	MRI	Radical hysterectomy	Identification of DSI
Yi et al. [57]	Ovarian cancer	Retrospective, single center study	71 + 31	RF, SVM, LASSO, ICC	Genomic data, CT	Platinum-based chemotherapy after maximal cytoreductive surgery	Platinum resistance
Lefebvre et al. [58]	Endometrial cancer	Retrospective, multi-center study	94 + 63	RF, Mann-Whitney U test, Mann-Whitney U test	MRI	Hysterectomy	DMI, LVSI, high-grade status
Laios et al. [59]	Ovarian cancer	Postoperative, single center study	209	Kaplan-Meier, Cox proportional hazard regression analysis, five different classifiers	Clinical data	Cytoreductive surgery	PFS, OS
Hwangbo et al. [60]	Ovarian cancer	Retrospective, multi-center study	1002	LR, RF, SVM, DNN, Kaplan-Meier, log-rank test	Clinicopathologic characteristics, surgical findings, details of chemotherapy, treatment response, survival outcomes	Platinum-based NACT	Platinum sensitivity
Barber et al. [61]	Ovarian cancer	Retrospective, multi-center study	291	NLP, LR, RF, SVM, XGBoost, gradient boosting machines	Discrete data predictors, natural language processing of full text reports	Debulking surgery	Postoperative complication, hospital readmission

PCC Pearson correlation coefficient, NCRT Neoadjuvant chemoradiotherapy, ICC Inter-class correlation coefficients, LASSO Least absolute shrinkage and selection operator, ET Extremely randomized trees, RFE Recursive feature elimination, NTCP Logistic normal tissue complication probability, SBE-SVM Sequential backward elimination support vector machine, DMFS Distant metastasis-free survival

### Application of AI in cervical cancer

Cervical cancer is one of the most prevalent tumors in female patients [62]. One hundred and fourteen studies have shown that imaging-based prognostic characteristics using radiomics and deep learning methods can help predict treatment response, prognosis, dose distribution, and target volume delineation in patients with cervical cancer [19, 50, 63].

#### Conventional radiomics in cervical cancer

Conventional radiomics has demonstrated great potential in predicting the response of cervical cancer to chemoradiotherapy (CRT) [26], neoadjuvant chemoradiotherapy (NCRT) [19], neoadjuvant chemotherapy (NACT) [31], concurrent chemoradiotherapy (CCRT) [64], brachytherapy [65], curative radiotherapy with adjuvant cisplatin [66], intensity modulated radiation therapy (IMRT) [67]. Among the studies included in this systematic review, 12 articles investigated the application of conventional radiomics methods in predicting treatment outcomes in patients with cervical cancer. Gui et al. [19] utilized radiomics features of pre-treatment T2-weighted MRI to predict pathological complete response (pCR) after NCRT in 183 patients with LACC and achieved an area under the curve (AUC) of 0.8 in an eight-fold cross validation. Tian et al. [42] constructed a combined model that incorporated radiomics features from CT images, FIGO staging, and the age of 277 patients with LACC treated with NACT to predict the response in a multi-center study. This model demonstrated superior predictive performance compared to a single radiomics model. Most articles have demonstrated that the random forest (RF) model exhibits superior predictive capabilities compared to other machine learning models. The RF model showed a high level of accuracy in predicting the treatment response in cervical cancer. Furthermore, a combined model was found to be superior to a single model in these studies. Two studies combined FIGO staging features to construct a predictive model and achieved good results. All the included studies focused on LACC. However, most of these studies did not specify the histological subtype of the cervical cancer under investigation. Only two studies explicitly stated that their models were developed specifically for squamous cell carcinoma. Future research should aim to develop predictive models tailored to other histological subtypes of cervical cancer to enhance personalized treatment strategies.

Twenty-two articles explored the application of conventional radiomics-based prognostic features in cervical cancer to predict the OS, recurrence, DFS, and LC. Nevertheless, external validation was absent in half of the studies included, with most relying solely on single-center retrospective datasets. Zhang et al. [54] developed a radiomics model using Multiparametric MRI to predict PFS and OS in locally advanced cervical squamous cell cancer. A total of 185 patients were recruited for this study. Although the model exhibited promising performance during internal testing, its robustness could not be established without external validation. Three studies extracted radiomics features from multi-sequence MRI, including T1-weighted imaging, T2-weighted imaging, and diffusion weighted imaging. Multi-sequence MRI enables comprehensive tumor characterization, thereby enhancing the development of prognostic prediction models. Liu et al. [44] showed that radiomics features based on multiparametric MRI could effectively predict DFS in patients with LACC after CCRT. Kawahara et al. [68] developed a model using pre-treatment T1- and T2-weighted MRI radiomics features to predict recurrence in patients with LACC who were undergoing radiotherapy. In an external validation cohort of 26 patients, the model achieved an AUC of 0.96, significantly outperforming the prediction models based on single-sequence MRI (AUC = 0.69). While multiple studies leveraged multi-center cohorts, only two investigations systematically addressed inter-site technical variations (e.g., scanner manufacturers and imaging parameters) by implementing ComBat harmonization (combining batch effects across technologies). Lucia et al. [69] extracted radiomics features from ^18^F-FDG PET and MRI to predict DFS and locoregional control (LRC) in LACC. This study incorporated data from 190 patients across three centers. The prediction models for DFS and LRC achieved > 90% accuracy in both external validation cohorts. These multi-center studies demonstrated robust predictive performance, further supporting the potential feasibility of radiomics for prognostic prediction in patients with cervical cancer. This systematic evaluation performed using the radiomics approach to predict the prognosis of cervical cancer revealed marked heterogeneity in the study quality across these articles. There remains a critical need for large-scale studies with prospective designs and independent external validations to strengthen clinical translatability.

Six studies revealed the potential predictive value of conventional radiomics for adverse events. Nevertheless, the predictive performance of these proposed models was suboptimal; two studies developed models with AUC values below 0.7, whereas the maximum AUC achieved across all studies was 0.85, indicating limited performance. Le et al. [20] utilized clinical characteristics and CT-derived radiomics features to predict acute severe hematologic toxicity in patients with cervical or endometrial cancer during radiotherapy, achieving an AUC of 0.85 in the external validation set. Additionally, Wei et al. [47] found that radiomics features could predict radiation proctitis. Yu et al. [48] found that the radiomics signature has the potential to predict postoperative malnutrition in patients with cervical cancer receiving postoperative radiotherapy with or without chemotherapy. Moreover, many studies have incorporated dose parameters into their toxicity prediction models, indicating that the adverse events associated with radiotherapy showed a significant correlation with radiation dose parameters.

#### Spatial-related unconventional radiomics in cervical cancer

**Fig. 3:**
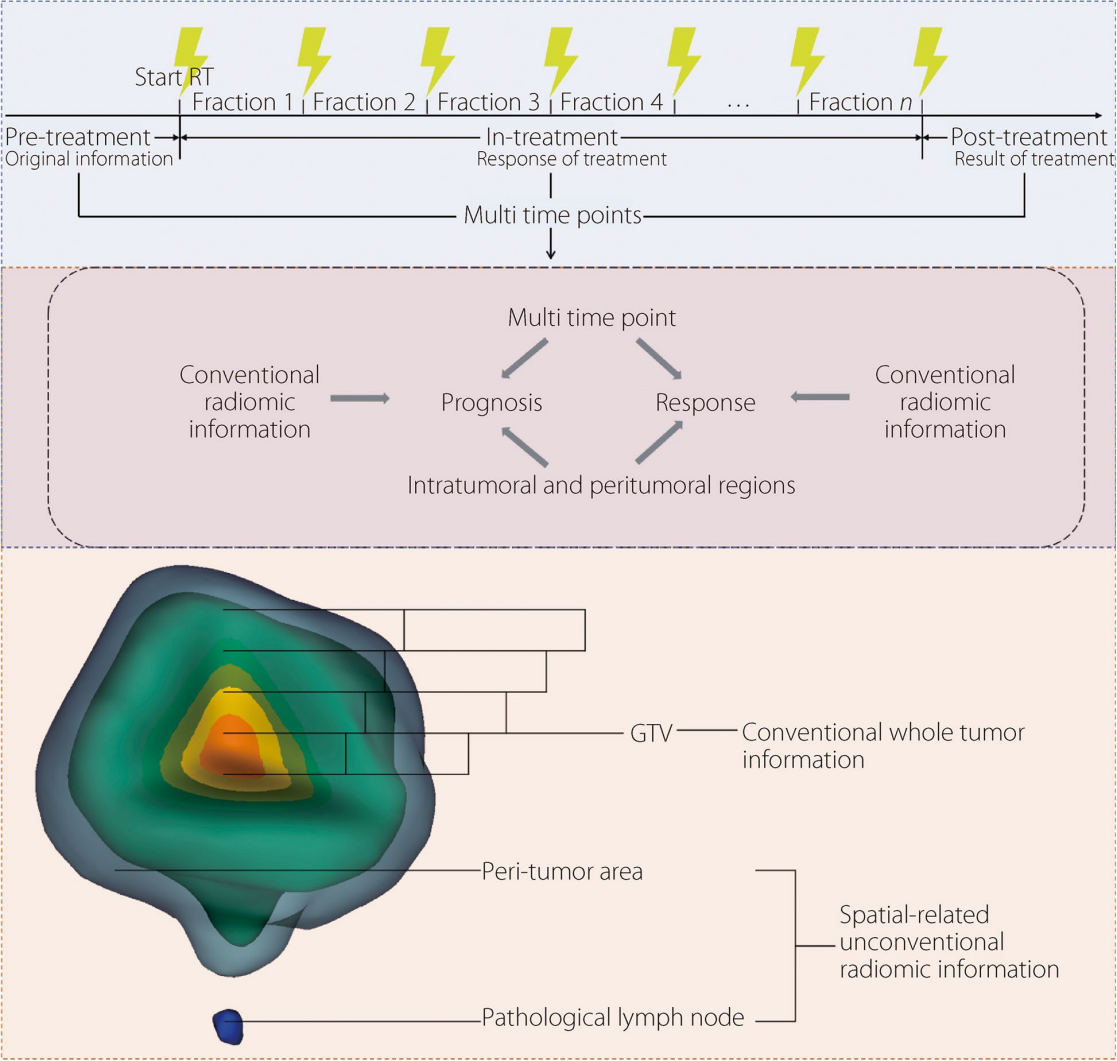
Radiomics features calculated from multi-time point or intratumoral and peritumoral regions can be used to predict prognosis and response

In contrast to conventional radiomics, eight studies employed radiomics features from both intratumoral and peritumoral regions to predict the treatment response and prognosis of cervical cancer [29, 50]. These studies demonstrate that incorporating radiomics features calculated from intratumoral and peritumoral regions and lymph nodes can improve model performance in comparison to using only radiomics features calculated from the tumor region (Fig. 3). For instance, Sun et al. [13] extracted radiomics features from the intratumoral region of T1-weighted and T2-weighted images and the peritumoral region of T2-weighted images before NACT for patients with LACC to predict clinical response. The combined model achieved an AUC of 0.999 in the external validation set across the three centers, which performed better than the radiomics models based on a single region. Wu et al. [51] extracted radiomics from intratumoral and peritumoral tissues on T2WI and ADC maps to predict lymph node metastasis (LNM) in cervical cancer. Combining radiomics features from intratumoral and peritumoral tissues on T2WI and clinical lymph node status, the model showed the best performance in predicting LNM, achieving an AUC of 0.847 in the validation set. Eight studies demonstrated that incorporating radiomics features from the peritumoral/lymph node regions could enhance the performance of the models in predicting the prognosis or clinical response in cervical cancer. This phenomenon may be attributed to the fact that peritumoral and lymph node radiomics features capture critical supplementary information regarding tumor microenvironment heterogeneity, invasive characteristics, and potential metastatic dissemination, which are not adequately represented by intratumoral features.

#### Temporal-related unconventional radiomics in cervical cancer

Similar to the radiomics features extracted from pre-treatment medical images to predict pCR, OS, and DFS, features extracted from medical images at multiple time points (pre-treatment, in-treatment, and post-treatment) have also been utilized to predict the prognosis [14] and treatment response [26, 53] of cervical cancer. Three studies investigated the potential of temporally related unconventional radiomics features for predicting treatment outcomes and prognosis in cervical cancer.

For example, Zhang et al. [14] utilized radiomics features extracted from primary lesions of pre-treatment and post-treatment axial T2WI MRI to predict DFS after CCRT in patients with LACC (AUC = 0.977). Additionally, Bowen et al. [53] extracted radiomics features of IB2-IVA cervical cancer patients based on MRI and PET scans at three time points to predict the response (AUC = 0.86). Cusumano et al. [26] applied radiomics features calculated based on MRI obtained at two time points (before treatment and mid-therapy) to predict pCR after CRT in patients with LACC, with an AUC of 0.77. Radiomics features based on multiple time points have high clinical value for response prediction and personalized treatment of patients with cervical cancer.

#### Deep learning in cervical cancer

In cervical cancer, deep learning-based approaches have also demonstrated great potential for predicting treatment response [31], recurrence [32, 54], rectum toxicity [33], bladder toxicity [55], deep stromal invasion (DSI) [56], dose distribution [70], and target volume delineation [71]. These deep learning models have demonstrated superior performance compared with conventional radiomics and statistical approaches, probably because of their ability to extract intricate, high-dimensional imaging features.

One major application of deep learning in cervical cancer is the prediction of the treatment response. Three articles included in the systematic review developed deep learning models to predict treatment responses in cervical cancer. For instance, Zhang et al. [31] developed a model incorporating MRI-derived DenseNet-121 and radiomics features to predict the response to NACT in 285 patients with LACC, achieving a strong predictive performance across multiple centers (AUC = 0.91). Furthermore, adverse responses associated with radiotherapy have attracted widespread attention. Huang et al. [72] utilized a transformer-based model combining CT imaging and clinical features to assess pelvic osteoporosis risk in patients undergoing radiotherapy, demonstrating significantly improved performance compared with conventional machine learning methods (AUC = 0.977). Cheon et al. [55] compared multiple AI approaches, including deep learning-based multilayer perceptron (MLP) and traditional statistical methods, to predict bladder toxicity in patients with cervical cancer undergoing radiotherapy. Their findings indicated that deep learning models outperformed conventional methods, suggesting their potential to improve personalized risk assessments. These studies highlight the advantages of deep learning models in capturing complex imaging patterns relevant to treatment responses.

Another important application is in the prediction of recurrence and metastasis. Five studies used deep learning models to predict the cervical cancer prognosis. Shen et al. [54] proposed a deep Learning framework based on 18F-FDG PET/CT to predict local recurrence and distant metastasis in patients with LACC receiving radiotherapy and achieved high predictive accuracy. Liu et al. [73] applied a vision transformer model to extract patch-level histopathological features from whole-slide images and subsequently utilized a recurrent neural network to predict LNM in 540 patients with cervical cancer. The model demonstrated strong generalizability in a prospective validation cohort (AUC = 0.91), further underscoring the effectiveness of deep learning for prognostic assessment.

Forty-seven studies explored the application of deep learning methods for automatic clinical target volume (CTV) and organs at risk (OARs) delineation during radiotherapy or brachytherapy in cervical cancer patients. Nie et al. [74] developed a deep learning network based on the U-net for CTV and OARs delineation in cervical cancer radiotherapy. In the test set, the automatic delineation of all target regions achieved satisfactory results (dice similarity coefficient: 0.84–0.93). The AI model was evaluated in a clinical setting. Three radiation oncologists applied the AI model to 20 new cervical cancer patients in an experiment. The clinical experiment achieved good results, and the AI model significantly reduced the time required by doctors to outline the targeted area (18.09 min *vs* 7.37 min for a doctor). Li et al. [75] applied the nn-Unet model to segment the CTV and OARs in 237 patients with cervical cancer undergoing high-dose-rate brachytherapy, and the model obtained high segmentation accuracy. These studies have demonstrated remarkable accuracy and reliability in automatically identifying target regions, offering significant potential for enhancing the precision and consistency of radiotherapy planning in cervical cancer. By automating the target delineation process, AI approaches can substantially reduce the time and effort required from clinicians.

Five studies used deep learning models for dose distribution prediction in radiotherapy, brachytherapy, and volumetric modulated arc therapy (VMAT) for patients with cervical cancer. Li et al. [70] proposed a multitask attention adversarial network (MtAA-NET) to predict the 3D radiotherapy dose distribution and auxiliary segment CTV and OARs based on the CT images of 42 patients of cervical cancer. The MtAA-NET model demonstrated superior predictive performance, outperforming the existing state-of-the-art models across almost all metrics. Zhang et al. [76] developed a 3D-Unet model to predict the dose distribution in VMAT for cervical cancer (*n* = 117) and tested its generalization for endometrial cancer (*n* = 20). The model showed excellent performance, with the mean absolute error for cervical cancer at 2.43% ± 3.17% and for endometrial cancer at 2.70% ± 3.54%. Although current studies have been conducted on small private datasets, they have shown promising results, highlighting the significant potential of deep-learning models for predicting radiotherapy dose distributions. Future research should validate these findings using larger datasets to further assess the robustness and generalizability of AI models for dose distribution prediction.

Collectively, these studies demonstrated the growing role of deep learning in cervical cancer prognosis and risk stratification. The superior performance of deep learning models over traditional approaches demonstrates their ability to capture intricate imaging features that are crucial for clinical decision making. Moreover, models that incorporate multi-modal data tend to achieve higher predictive accuracy, emphasizing the importance of integrating multi-modal information to enhance model robustness.

### Application of AI in ovarian cancer

Ovarian cancer is one of the most prevalent and aggressive gynecological malignancies worldwide. Ten articles used AI methods to predict platinum sensitivity [57, 60], prognosis [59], and postoperative complications [70] in ovarian cancer. In most studies, traditional radiomics approaches have been applied to ovarian cancer.

Seven studies developed a traditional machine learning model to predict the prognosis and platinum sensitivity in patients with ovarian cancer. Yi et al. [57] built a radiomic model by combining genomic data (human sulfatase 1) and CT radiomics features to predict platinum resistance in patients with ovarian cancer undergoing chemotherapy (AUC = 0.967). Their study revealed that the combination of genomic biomarkers and radiomics features significantly improved the predictive performance of the model. Laios et al. [59] assessed the performance of various machine Learning algorithms to predict the 2-year prognosis of ovarian cancer patients based on clinical data. They found that the support vector machine (SVM) and ensemble subspace discriminant algorithms achieved superior results in terms of accuracy indices compared to the logistic regression algorithm.

Two studies developed deep learning models to predict the response and prognosis of ovarian cancer. Hwangbo et al. [60] developed deep learning-based models based on clinical data to predict platinum sensitivity in patients with ovarian cancer using four classification algorithms, including a deep neural network (DNN). They found that the LR-based model performed the best (AUC = 0.741). Barber et al. [61] utilized an NLP model to select features from unstructured clinical records and employed several machine Learning models to predict 30-day unplanned hospital readmission in patients with ovarian cancer undergoing surgery, achieving an AUC of 0.70.

### Application of AI in endometrial cancer

Endometrial cancer is a tumor originating in the endometrium, and its prevalence continues to increase worldwide [77]. Ten studies explored the potential value of AI methods in the diagnosis [58], prognosis [78], and adverse events [20] of endometrial cancer.

Lefebvre et al. [58] built a 3D radiomics-based model by analyzing multiparametric MRI to predict deep myometrial invasion (MI) and lymphovascular space invasion (LVSI) in endometrial cancer patients. The AUCs of models for predicting MI and LVSI were 0.81 and 0.80 in an external validation set of 63 patients, respectively. Li et al. [78] built a model incorporating radiomics features extracted from T2-weighted MRI and clinical characteristics to predict the survival time of patients with endometrial cancer (AUC = 0.727). They found that an MRI-based radiomics signature provided incremental prediction values for survival time. Le et al. [20] utilized CT-based radiomics features to predict acute severe hematologic toxicity in patients with cervical and endometrial cancers undergoing radiotherapy (AUC = 0.85). Among these studies, nine articles validated the performance of the AI prediction model in an external validation set with robust results. These consistent results across independent cohorts strongly support the potential of AI for the treatment of endometrial cancer.

## Impact of different AI approaches on AI-assisted treatment in gynecologic oncology

**Fig. 4:**
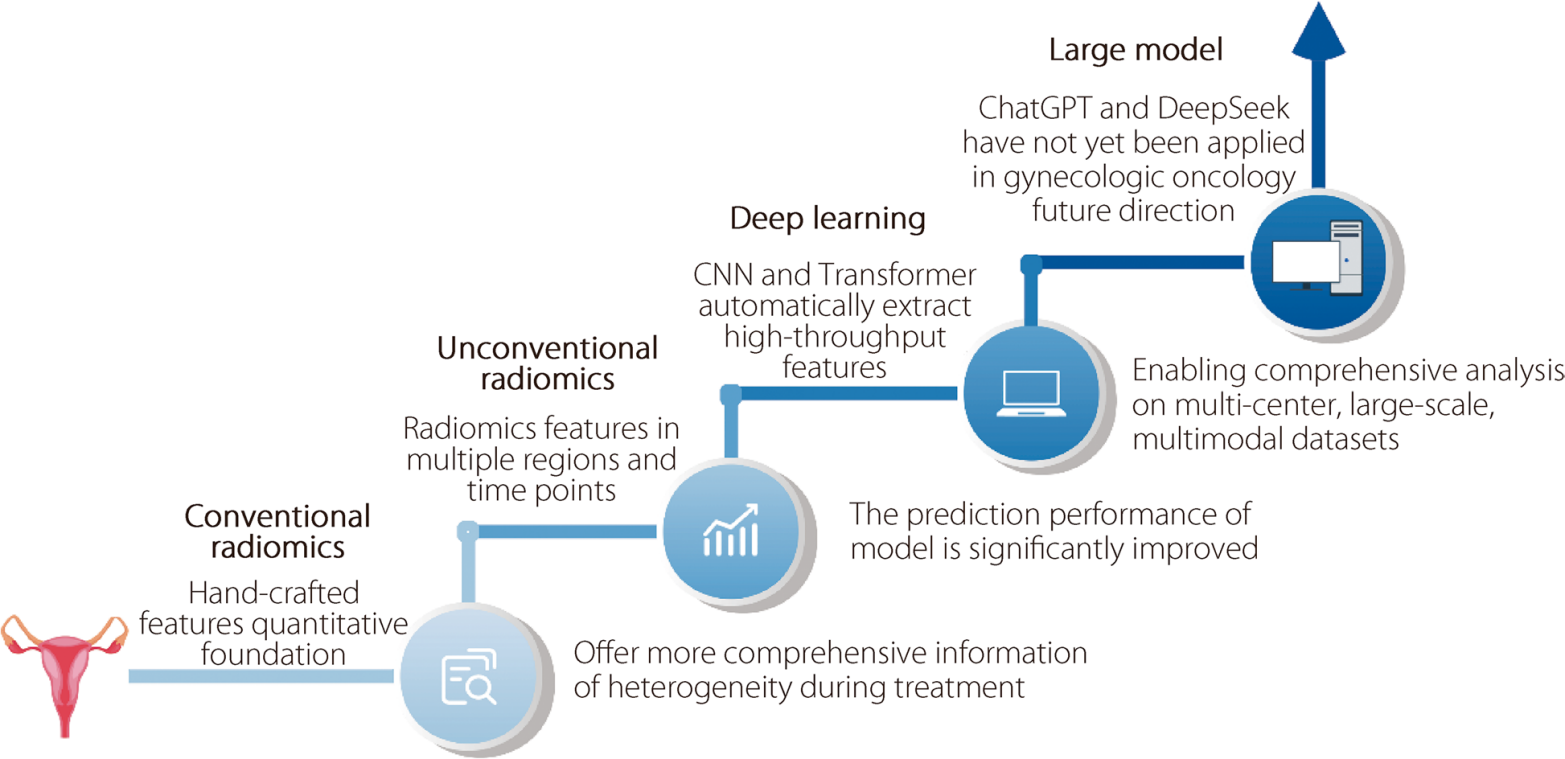
From radiomics to large model: The evolutionary landscape of AI-assisted treatment in gynecologic oncology

The application of AI in gynecological oncology has shown remarkable progress driven by various approaches that address the complex demands of assisting doctors with treatment, including treatment response prediction, prognosis prediction, adverse event prediction, dose prediction, and CTV delineation (Fig. 4).

Conventional radiomics extracts handcrafted features from medical images, enabling a quantitative approach for treatment assessment. This method provides a solid foundation for AI-assisted treatment in gynecological oncology, demonstrating the feasibility of identifying predictive indicators. However, their performance is often constrained by their limited ability to capture complex and high-dimensional imaging features. To address these limitations, spatial-related unconventional radiomics analyzes multiple ROI regions, offering more comprehensive characteristics of heterogeneity in gynecologic tumors, whereas temporal-related unconventional radiomics utilized longitudinal imaging data to monitor dynamic changes during gynecologic cancer treatment. Although these advanced radiomics techniques have enhanced predictive performance, they rely on manual feature analysis and are sensitive to imaging variability.

In contrast, deep learning methods, particularly CNN and transformer models, have improved AI-assisted treatment in gynecologic oncology by automatically learning features from images. Deep-learning models generally outperform radiomics methods because of their ability to capture high-throughput and multiscale information. Moreover, deep-learning models that integrate clinical data and multi-modal imaging offer more accurate and personalized predictions, leading to significant improvements in response prediction, prognosis assessment, dose prediction, and target delineation in gynecologic oncology.

Although large language models (LLMs), such as ChatGPT and DeepSeek, have not yet been explored for AI-assisted treatment of gynecologic cancer, their rapid development and proven success in NLP and multimodal tasks demonstrate their great potential in the medical field. LLMs trained on vast datasets can understand complex medical concepts and generate insightful analyses, making them well-suited for integrating multi-source data, providing personalized treatment recommendations, and supporting clinical decision-making in gynecologic oncology. Compared to traditional deep-learning models, LLMs can efficiently handle vast, heterogeneous datasets, offering a more scalable and generalizable solution. In the future, applying large models in gynecologic oncology could overcome the current limitations by enabling comprehensive analysis of multi-center, large-scale datasets, facilitating accurate, robust, and clinically applicable AI-assisted treatment. The progression from traditional radiomics to deep learning and LLMs has significantly enhanced the precision and robustness of AI-assisted treatments in gynecologic oncology. Deep learning methods, with their automatic feature extraction, have outperformed traditional approaches in many clinical tasks, whereas large models offer promising advantages in handling large datasets for personalized medicine. AI-assisted treatments are expected to play an increasingly significant role in the management of gynecological cancers.

## Evaluation of AI studies

With the development of radiomics and deep learning, several guidelines and evaluation frameworks have been developed to assess AI research in medical imaging. Radiomics Quality Score (RQS) is a widely accepted evaluation standard in radiomics [59]. Nevertheless, the definitions of certain RQS items may be open to interpretation, potentially compromising the repeatability of scores, even among specialists in the field. Additionally, several guidelines and scores have been proposed for evaluating AI research on medical images, including the METhodological RadiomICs score (METRICS) [79], CheckList for Artificial Intelligence in Medical Imaging (CLAIM) [80], and others [81–83].

### RQS

The RQS is a commonly recognized evaluation criterion in the field of radiomics research proposed by Lambin et al. [84]. The RQS of eight radiomics studies [19, 20, 31, 46, 51, 57, 85, 86] were calculated. The eight representative papers covered the three AI approaches mentioned above: deep Learning, conventional radiomics, and unconventional radiomics. These eight articles were centered on cervical, ovarian, and endometrial cancers. The average RQS of these studies was 12, with the highest value [20, 31], 18 and the lowest value [85, 86], 5. Most studies that lacked external validation exhibited low RQS scores, indicating that the models used in these studies require further validation for clinical application. There is a notable lack of prospective studies that validate models for predicting clinical outcomes. Most of the aforementioned radiomics studies lack an explanation for the relationship between radiomics features and molecular biology. Few studies have conducted phantom analyses to evaluate the impact of different scanners and vendors on AI models, which may limit the generalizability of radiomics or deep-learning models across centers. Moreover, half of the studies failed to provide an open-source code, limiting the reliability and reproducibility of the models.

### METRICS

METRICS is a quality scoring tool that assesses the quality of radiomics studies, proposed by Kocak et al. [79]. The studies were classified into five categories (very low, low, moderate, good, and excellent) based on the METRICS scoring system. Kocak et al. designed a web application to assist in calculating METRICS scores and it was used to calculate the METRICS scores of eight radiomics studies [19, 20, 31, 46, 51, 57, 85, 86]. The eight articles under consideration were identical to those evaluated using the RQS scores presented above. Two of the articles were of excellent grade [20, 31], while the remaining articles were graded as good. The highest MET score was 91.5% [20], while the lowest was 67.1% [86], with a mean of 75.3% across the eight articles. Most studies scored zero for the option “Clinical translatability of imaging data sources for radiomics analysis,” indicating a lack of clinical translation of AI for treatment response prediction in gynecological oncology. Furthermore, the majority of studies exhibit inferior performance in terms of “data availability,” “code availability,” “model availability,” and “external testing,” which significantly limits the reliability and generalizability of the research.

### CLAIM

A CLAIM was proposed by Mongan et al. [80] to standardize the application of AI in medical images. Forty-two items were included in the CLAIM checklist related to the data, ground truth, model, training, and evaluation. The CLAIM checklist is a tool used by authors and reviewers to improve the quality of AI manuscripts. Wang et al. [87] utilized CLAIM to evaluate the quality of research utilizing a deep learning model for the segmentation of lung cancer in CT images.

## Clinical translatability of AI models in gynecologic tumors

The clinical translatability of AI models in oncology has been widely studied. Determining how to apply AI models in clinical practice in oncology remains challenging.

The clinical translatability of AI research in gynecological oncology is currently in its primary stage. Nevertheless, the significance of clinical translatability has been emphasized in many articles [88, 89]. The clinical translatability of AI models in gynecologic oncology is expected to become more concentrated in the future. Notably, our discussion of clinical translatability draws primarily from non-gynecological cancers because of the current scarcity of radiomics/AI implementations, specifically for gynecologic oncology. This underscores both a critical knowledge gap and an opportunity for future translational studies on cervical, ovarian, and endometrial cancers.

In addition, several studies have focused on the clinical translatability of AI models to other cancers. Several studies have enabled physicians to use AI models for decision-making, thereby validating the clinical translatability of these models. Six resident trainees reviewed images of the same patient to diagnose cervical LNM for thyroid cancer using a deep learning model [90]. The results showed that the overall diagnostic confidence Levels increased significantly from 3.90 to 4.30 with the assistance of the deep learning model. Two radiologists used a deep learning model to diagnose benign and malignant lesions of breast nodules in ultrasound images [91]. Significant improvements in AUC, sensitivity, and specificity were observed for inexperienced radiologists (0.547 *vs* 0.800, 50.00% *vs* 78.57%, 59.30% *vs* 81.40%, respectively) compared with those without the assistance of deep Learning models. Furthermore, the rate of unnecessary Biopsies decreased by 24% among the inexperienced radiologists. These studies indicate that deep learning models can aid doctors in clinical decision making.

Various AI devices for medical imaging have been approved by the Food and Drug Administration (FDA) (https://www.accessdata.fda.gov/scripts/cdrh/cfdocs/cfpmn/pmn.cfm). The Avenda Health AI Prostate Cancer Planning Software was approved by the FDA in 2022 and, which was designed to identify suspicious Lesions for prostate cancer. The device generates a probability value for each voxel within the prostate gland, which indicates the probability of prostate cancer. This information is used to generate a cancer map. A Cardiac CT Function Software Application was designed to detect and segment heart structures to assist physicians in visualizing and evaluating cardiac function of patients, and was approved by the FDA in 2024. The software provides a function to semi-automatically segment the heart and calculate cardiac function metrics such as the ejection fraction. ProFound AI V3.0, a software approved by the FDA in 2021, was developed to identify suspicious breast Lesions. In 2021, Komatsu et al. [92] reviewed the clinical applications of AI in ultrasound imaging. This article mentioned numerous medical AI devices approved by the FDA for imaging different body areas, including the breast, thyroid, bladder, and heart. This demonstrates that AI devices have the potential for use in clinical practice. Nevertheless, the majority of the AI software approved by the FDA for processing medical images has been developed for diagnosis rather than for predicting the response or prognosis of treatment. It is hoped that more AI software will be designed in the future to predict treatment outcomes or patient prognosis.

The clinical translatability of AI models for predicting response and prognosis in gynecologic tumors has significant limitations, and it is unclear whether any studies directly apply AI models in the clinical practice of gynecological oncology. There are also limitations to the clinical translatability of AI models to other fields of oncology. Bakrania et al. [93] mentioned the limitations and challenges in the clinical translatability of AI models in the field of liver cancer, and emphasized the importance of achieving ultimate clinical applicability. Patel et al. [94] analyzed radiomics studies of meningiomas and found that few articles evaluated the clinical applicability of radiomics models. The limited clinical translatability of AI models in the field of oncology may be because of their low interpretability of AI models, which makes doctors wary of the results generated by AI models. Research should explore the biological/histopathological correlates of imaging phenotypes to improve the clinical translatability of AI models [95].

## Discussion

Recently published studies have shown the potential of AI in discovering prognostic indicators associated with assisting the treatment of gynecologic tumors. However, the RQS of these studies was relatively low, and there are still some issues and concerns regarding the clinical translatability of these AI-enabled prognostic indicators.

Recent studies demonstrated that integrating quantitative features from intra- and peritumoral regions and multiple time-point medical images into a unified AI model can enhance the performance of AI-enabled prognostic indicators. Chen et al. [96] demonstrated a strong positive correlation between tumor infiltration levels of certain endometrial cancer cells and the expression of TMEM150B, SIGLEC1, and CTSW. Their study showed that clinical outcomes were influenced by the composition of the tumor microenvironment. During the treatment process, gynecologic tumors undergo dynamic changes that can be reflected in multiple time-point medical images. Consequently, conducting quantitative analysis of medical images at different time points holds great promise for improving the predictive performance of AI models in assessing treatment outcomes. However, most current AI methods for gynecologic tumors utilize simple concatenation operations to merge features from multiple ROIs and time-point medical images [52, 53]. In the future, it is imperative to explore more sophisticated and efficient feature fusion methods to further enhance the performance of AI models for gynecologic tumors.

Moreover, current published studies have typically validated AI-enabled prognostic characteristics using single-center and retrospective cohorts. It is important to note that medical images exhibit variations due to differences in acquisition parameters and scanners, leading to potential variability in the performance of AI-enabled prognostic characteristics on medical images from different hospitals. Therefore, it is crucial to validate these prognostic characteristics in multi-center prospective cohorts.

CNN models are widely used in studies predicting the treatment response and prognosis for gynecologic tumors; however, research utilizing transformer models in this field is limited. The transformer relies entirely on self-attention mechanisms to calculate the representation between inputs and outputs. The transformer model has an encoding-decoding structure that consists of multi-headed self-attention [97]. The self-attention mechanism enables the model to assess the significance of various elements in the input sequence and to dynamically modulate their influence on the output. Compared to CNN, transformers have the following advantages. First, because of its self-attention mechanism and positional embedding, the transformer can capture long-range dependencies in sequential data and global information in images. In addition, the transformer-based model is suitable for simultaneously processing various types of data, including text and images. Transformer-based models have achieved great success in NLP and computer vision, such as in ChatGPT, Bert, and GLM-130B [98–100]. Additionally, transformer-based models have been widely employed in medical image analysis [101]. In previous studies, transformer models have been exploited for the segmentation, classification, and reconstruction of medical images. In the future, the application of the transformer model in predicting the treatment response and prognosis of gynecologic tumors should be further explored.

Most AI-enabled prognostic characteristics of gynecologic tumors have been developed using hundreds of well-annotated cases [13, 42, 49]. However, as the complexity of the AI model and the number of imaging features increase, the risk of overfitting also increases, causing the model to perform well in the training cohort, but poorly on unseen data. Therefore, large-scale medical imaging is required to develop a stable and generalized prognostic model. Given the scarcity of well-annotated medical data and the abundance of unannotated medical data, developing AI models using semi-supervised or self-supervised learning methods may be an effective alternative, which suggests that unannotated data can also contribute to improving the performance of AI models [102]. Currently, large models, such as RETFound (a foundation model for retinal images) [103] and Generalist Medical AI [104] are usually trained through self-supervised learning on extensive image data to learn rich and accurate image representations. These models have excelled in computer vision applications. With extensive medical data, the exploration of a transformer-based large-foundation model for gynecologic tumors is warranted.

The clinical translatability of AI analyses in gynecological oncology remains significantly constrained. Both the RQS and METRICS scores indicate that only a limited number of studies have assessed the clinical applicability of their models. Furthermore, there is a paucity of research addressing the biological correlates of AI-generated results and only a few studies have conducted cost-effectiveness analyses. These limitations adversely affect the clinical translatability of AI models in gynecological oncology. The potential of AI models will only be realized when their clinical translatability is validated. Future research should focus on enhancing the clinical applicability of AI models.

## Conclusions

In conclusion, AI methods have been widely applied in gynecological oncology and have demonstrated significant potential for discovering prognostic characteristics. However, several issues and concerns must be addressed before they can benefit clinical decision-making for patients with gynecologic tumors, particularly the limited clinical translatability of the AI model. These AI models can provide substantial benefits to patients in clinical practice, but only when the challenges associated with clinical translation are resolved.

## Data Availability

Not applicable.

## Abbreviations

AI: Artificial intelligence
MRI: Magnetic resonance imaging
CT: Computed tomography
PET: Positron emission tomography
ADC: Apparent diffusion coefficient
LACC: Locally advanced cervical cancer
NLP: Natural language processing
ROI: Region of interest
GTV: Gross tumor volume
FDG: Fluorodeoxyglucose
OS: Overall survival
PFS: Progression-free survival
CNN: Convolutional neural network
CRT: Chemoradiotherapy
NCRT: Neoadjuvant chemoradiotherapy
NACT: Neoadjuvant chemotherapy
CCRT: Concurrent chemoradiotherapy
IMRT: Intensity modulated radiation therapy
pCR: Pathological complete response
DFS: Disease-free survival
LNM: Lymph node metastasis
MLP: Multilayer perceptron
AUC: Area under the curve
SVM: Support vector machine
DNN: Deep neural network
RQS: Radiomics quality score
FDA: The Food and Drug Administration
LC: Local control
VGG: Visual geometry group
LRC: Locoregional control
CTV: Clinical target volume
OAR: Organ at risk
VMAT: Volumetric modulated arc therapy
MtAA-NET: Multitask attention adversarial network
MI: Myometrial invasion
LVSI: Lymphovascular space invasion
LLM: Large language model
CLAIM: CheckList for Artificial Intelligence in Medical Imaging
METRICS: METhodological RadiomICs Score
PCC: Pearson correlation coefficient
RF: Random forest
ICC: Inter-class correlation coefficients
DSI: Deep stromal invasion
LASSO: Least absolute shrinkage and selection operator
ET: Extremely randomized Trees
RFE: Recursive feature elimination
NTCP: Logistic normal tissue complication probability
SBE-SVM: Sequential backward elimination support vector machine
DMFS: Distant metastasis-free survival
